# Integrating Proteomics and GWAS to Identify Key Tissues and Genes Underlying Human Complex Diseases

**DOI:** 10.3390/biology14050554

**Published:** 2025-05-16

**Authors:** Chao Xue, Miao Zhou

**Affiliations:** 1 Medical College, Jiaying University, Meizhou 514031, China; 2 Zhongshan School of Medicine, Sun Yat-sen University, Guangzhou 510080, China

**Keywords:** genome-wide association study, proteomics, disease-associated tissues, complex diseases, associated genes, fine-mapping

## Abstract

Background: The tissues of origin and molecular mechanisms underlying human complex diseases remain incompletely understood. Previous studies have leveraged transcriptomic data to interpret genome-wide association studies (GWASs) for identifying disease-relevant tissues and fine-mapping causal genes. However, according to the central dogma, proteins more directly reflect cellular molecular activities than RNA. Therefore, in this study, we integrated proteomic data with GWAS to identify disease-associated tissues and genes. Methods: We compiled proteomic and paired transcriptomic data for 12,229 genes across 32 human tissues from the GTEx project. Using three tissue inference approaches—S-LDSC, MAGMA, and DESE—we analyzed GWAS data for six representative complex diseases (bipolar disorder, schizophrenia, coronary artery disease, Crohn’s disease, rheumatoid arthritis, and type 2 diabetes), with an average sample size of 260 K. We systematically compared disease-associated tissues and genes identified using proteomic versus transcriptomic data. Results: Tissue-specific protein abundance showed a moderate correlation with RNA expression (mean correlation coefficient = 0.46, 95% CI: 0.42–0.49). Proteomic data accurately identified disease-relevant tissues, such as the association between brain regions and schizophrenia and between coronary arteries and coronary artery disease. Compared to GWAS-based gene association estimates alone, incorporating proteomic data significantly improved gene association detection (AUC difference test, *p* = 0.0028). Furthermore, proteomic data revealed unique disease-associated genes that were not identified using transcriptomic data, such as the association between bipolar disorder and *CREB1*. Conclusions: Integrating proteomic data enables accurate identification of disease-associated tissues and provides irreplaceable advantages in fine-mapping genes for complex diseases.

## Simple Summary

Understanding the root causes of complex human diseases, such as schizophrenia, rheumatoid arthritis, and heart disease, remains a major challenge. Scientists have often used genetic information and RNA data to identify which tissues and genes are involved in these diseases. However, proteins play a more direct role in biological processes than RNA. In this study, we integrated large-scale protein data with genetic studies to better identify the specific tissues and genes linked to six common diseases. We found that protein information helped pinpoint more accurate disease-relevant tissues—such as coronary arteries in coronary artery disease—and uncovered important genes that RNA-based methods missed. For example, a gene called *CREB1* was linked to bipolar disorder based on protein data but not RNA data. Importantly, we also found that integrating protein and RNA data improves the identification of disease-related genes and biological pathways. This highlights the essential role of proteomics in uncovering the genetic mechanisms behind complex diseases.

## 1. Introduction

Although genome-wide association studies (GWASs) have identified numerous genetic variants associated with complex diseases, understanding their biological functions and elucidating the underlying pathogenic mechanisms remain largely unresolved [1]. It is still unclear in which tissues these genetic variants exert their pathogenic effects and which genes they influence [2,3].

The development and accumulation of multi-omics data, particularly transcriptomic data, have facilitated efforts to address these questions [4]. Several statistical methods have been developed to integrate GWAS signals with molecular data at the tissue or cellular level to infer disease-associated tissues and genes. For instance, S-LDSC [5] infers relevant tissues based on the heritability enrichment of tissue-specific genes, MAGMA [6] integrates gene-level association statistics with gene expression data across different tissues to estimate tissue associations, and DESE [2] leverages the tissue-specific expression patterns of disease-associated genes to identify relevant tissues while also enabling fine-mapping of associated genes.

However, existing approaches rely exclusively on transcriptomic data to interpret GWAS findings, with a notable absence of proteomic-based analyses. This is likely due to the limited availability of high-quality quantitative proteomic data [7]. Nevertheless, according to the central dogma, proteins more directly reflect cellular molecular activities than RNA, and studies have shown that protein and RNA expression levels exhibit significant differences [7,8,9]. Therefore, integrating proteomic data with GWAS to identify disease-associated tissues and genes is an urgent and important step in understanding the mechanisms of complex diseases.

**Figure 1:**
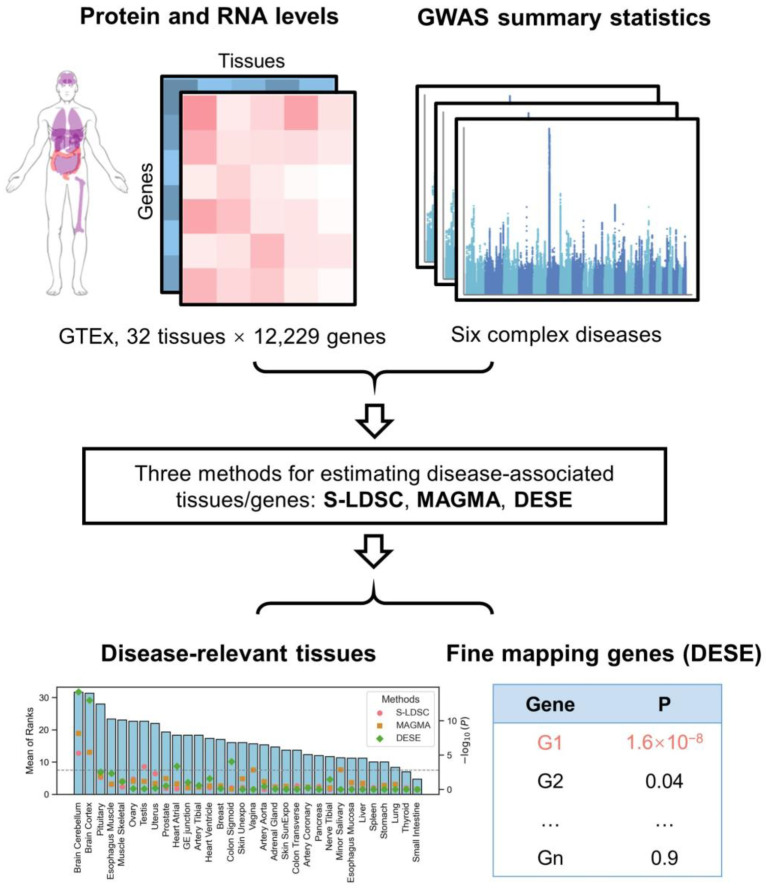
Overview of the analytical workflow.

To address this gap, we compiled proteomic and paired transcriptomic expression profiles from 32 normal human tissues in the GTEx project [7]. Using three widely adopted approaches [2,5,6] for identifying disease-associated tissues and genes, we analyzed GWAS data for six representative complex diseases. We systematically evaluated and compared the effectiveness of using proteomic profiles to infer disease-associated tissues and genes (Figure 1), highlighting the irreplaceable role of proteomics in deciphering the mechanisms of complex diseases.

## 2. Materials and Methods

### 2.1. Paired Protein and RNA Expression Profiles

We obtained paired protein and RNA expression profiles for 32 tissues from a study that quantified the proteome of normal human tissues in the GTEx project [7]. Protein expression data were extracted from Table S2 (E sheet) of the original study [7], which reports the median relative protein abundance for each tissue type. RNA expression data were obtained from Table S3 (B sheet) [7], which provides median RNA levels for protein-RNA co-quantified genes. To facilitate subsequent tissue and gene association analyses, we converted gene identifiers from Ensembl Gene IDs to HGNC gene symbols, ultimately retaining 12,229 genes for further investigation.

### 2.2. GWAS Summary Statistics

We collected GWAS summary statistics for six representative complex diseases (Table 1), including two psychiatric disorders (bipolar disorder [10] and schizophrenia [11]), one cardiovascular disease (coronary artery disease [12]), two immune-related diseases (Crohn’s disease [13] and rheumatoid arthritis [14]), and one metabolic disorder (type 2 diabetes [15]). The average sample size across these GWAS datasets was about 260 K. All GWAS datasets were derived from European ancestry populations, and we used Phase 3 of the 1000 Genomes Project (European population) as the reference panel to calculate linkage disequilibrium (LD) in subsequent analyses. Due to the complexity of LD patterns and genetic structure, the major histocompatibility complex (MHC) region was excluded from all analyses.

### 2.3. Tissue Correlation Analysis

We applied the robust-regression z-score (REZ) method [2] to compute tissue-specific expression at the protein level, expressed as Z-scores. Based on these Z-scores, we calculated Spearman correlation coefficients to assess similarity between different tissues. To evaluate the correlation between protein abundance and RNA expression, we computed Spearman correlation coefficients for tissue-specific expression levels of protein and RNA within the same tissues.

### 2.4. Identification of Disease-Associated Tissues

We used the following three methods to infer disease-associated tissues: S-LDSC [5], MAGMA [6], and DESE [2]. S-LDSC (version v1.0.1, https://github.com/bulik/ldsc, accessed on 2 March 2025) was applied with the top 1000 tissue-specific genes for stratified heritability enrichment analysis. MAGMA (version v1.10, https://cncr.nl/research/magma, accessed on 2 March 2025) was used with the “--gene-covar” parameter specified for tissue-specific expression profiles in tissue association analyses. DESE was implemented in KGGSEE v1.1 (https://pmglab.top/kggsee, accessed on 2 March 2025), with a conditional gene-based association analysis threshold of false discovery rate (FDR) < 0.05 and a maximum of 1000 genes. To integrate tissue association estimates from the three methods, we calculated the mean rank as the combined metric.

### 2.5. Fine-Mapping of Disease-Associated Genes

DESE not only identifies disease-associated tissues but also performs fine-mapping of disease-relevant genes through conditional gene-based association analysis. Specifically, it builds upon the effective chi-squared (ECS) [16] framework to compute gene-level association statistics from GWAS SNP-level summary data. The conventional *p*-value-based method (i.e., conditional ECS) conducts conditional analysis using these gene-based *p*-values alone, without incorporating external information. In contrast, DESE integrates tissue-specific expression data—either at the RNA or protein level—as an additional layer of functional information to guide the conditional analysis. By leveraging both gene-based association statistical signals and functional specificity, DESE enables the identification of distinct sets of candidate causal genes under different omic contexts.

To integrate the fine-mapped genes derived from RNA-based DESE and those from protein-based analyses, we calculated the geometric mean of the gene-level *p*-values from both sources. The resulting value was considered the integrated gene-level *p*-value. This integration approach assumes that both RNA-level and protein-level evidence contribute complementary information toward the gene’s involvement in disease, and the geometric mean provides a conservative yet balanced way to combine significance levels without being overly influenced by extreme values.

The integrated *p*-value was calculated as follows:

(1) $$P_{integrated}=\sqrt{P_{RNA}\times P_{Protein}}$$

where $P_{RNA}$ is the gene-level *p*-value from RNA-based DESE and $P_{Protein}$ is the gene-level *p*-value from protein-based DESE.

In the Results section of this study, for clarity, we use “*p*-value” to refer to the traditional *p*-value-based conditional association analysis (i.e., conditional ECS), and “RNA” or “Protein” to denote DESE analyses that incorporate RNA expression or protein abundance, respectively. “RNA + Protein” denotes the integrated *p*-value derived from both RNA- and protein-based DESE analyses (see Equation (1)). For all strategies, we defined a gene as significantly associated with the disease if it met the FDR threshold of <0.05.

### 2.6. Evaluation of Disease-Associated Genes

We compared the performance of gene fine-mapping using the abovementioned strategies. To evaluate the associations between diseases and genes, we used the PubMed web API (https://eutils.ncbi.nlm.nih.gov/entrez/eutils/esearch.fcgi, accessed on 15 March 2025) to search for publications where both the disease and gene appeared in the title or abstract, defining a disease-gene association as positive if the number of relevant publications was ≥5. For each disease, we computed the area under the ROC curve (AUC) for different methods and tested AUC differences across all diseases using a paired two-tailed *t*-test.

### 2.7. Functional Enrichment Analysis

We performed Gene Ontology (GO) [17,18] and KEGG pathway [19] enrichment analysis on disease-associated genes using the g:Profiler interface (https://biit.cs.ut.ee/gprofiler/gost, accessed on 19 March 2025) [20]. The GO covers the following three categories: Biological Process (BP), Cellular Component (CC), and Molecular Function (MF). The *p*-value correction method employed was the g:SCS algorithm provided by g:Profiler, with a significance threshold of corrected *p* < 0.1.

### 2.8. Protein-Specific Disease-Associated Gene Analysis

We focused on identifying unique disease-gene associations that could be detected using proteomic expression profiles as compared to transcriptomic data. We compared the normalized ranks of tissue-specific expression at both the protein and RNA levels for protein-specific disease-associated genes in disease-associated tissues, aiming to identify genes that show low tissue-specific expression at the RNA level but high tissue-specific expression at the protein level. Protein-specific disease-associated genes were defined as those that met the following criteria: (i) statistically significant in the protein-based fine-mapping analysis (FDR < 0.05) and (ii) not significant in the RNA-based analysis (FDR > 0.05).

To evaluate the difference in tissue-specific expression of genes between RNA and protein levels, we calculated a normalized rank based on the tissue-specific Z-score (calculated by REZ for each gene). Specifically, we ranked all genes within each tissue according to their Z-scores, separately for RNA and protein data. For each gene *g* in tissue *t*, the normalized rank $r_{g,t}^{\left(X\right)}$ at level *X* (RNA or protein) was calculated as follows:

(2) $$r_{g,t}^{\left(X\right)}=\frac{{rank}_{g,t}^{\left(X\right)}}{N}$$

where ${rank}_{g,t}^{\left(X\right)}$ is the position of gene *g* in the ascending Z-score ranking (i.e., rank 1 indicates the lowest tissue specificity) in tissue *t*, and N is the total number of genes included in the analysis. A higher normalized rank indicates higher tissue specificity. This approach enables a direct and comparable measurement of tissue specificity between RNA expression and protein abundance.

### 2.9. Analysis Code

The code for all analyses conducted in this study is publicly available at https://github.com/chaoxue-gwas/pDESE (accessed on 6 April 2025).

## 3. Results

### 3.1. Characteristics of Tissue-Specific Protein Expression

Compared to original gene expression levels, tissue-specific gene expression more sensitively reflects the biological characteristics of tissues, as it eliminates the influence of housekeeping genes that are highly expressed across all tissues [2]. Therefore, we first calculated tissue-specific expression for subsequent analyses.

**Figure 2:**
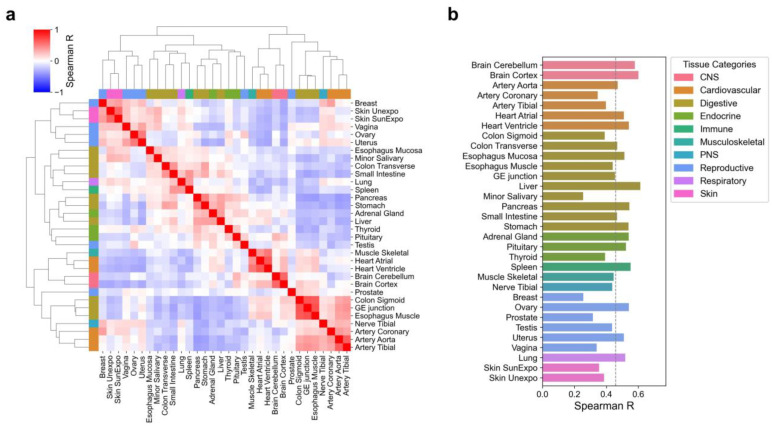
Tissue correlation according to protein abundance. (**a**) Heatmap of Spearman correlation coefficients between tissues, calculated based on tissue-specific protein abundance. (**b**) Spearman correlation coefficients between tissue-specific protein abundance and RNA expression within the same tissue. The dashed line represents the mean value. Different colors represent tissue categories, where CNS denotes the central nervous system and PNS denotes the peripheral nervous system.

We assessed the correlation coefficients between tissues based on protein-level tissue-specific expression (Figure 2a). Tissues with similar biological functions exhibited higher correlations. For example, the brain cortex and cerebellum showed a strong correlation (r = 0.60). In contrast, the brain cortex’s correlation with other tissues ranged from −0.34 to 0.27. Similarly, the correlation between the ventricles and atria of the heart was 0.66, the correlation between the ventricles and skeletal muscle was 0.53, while the correlation between the ventricles and other tissues ranged from −0.35 to 0.27. These results indicate that tissue-specific protein expression effectively captures tissue-specific biological properties.

We further compared the correlation between tissue-specific expression at the protein and RNA levels within the same tissue (Figure 2b). Overall, moderate correlations were observed, with an average correlation coefficient of 0.46 (95% CI: 0.42–0.49). The highest correlation was observed in the liver (r = 0.61), while the lowest correlation was observed in minor salivary glands (r = 0.25).

### 3.2. Disease-Associated Tissues

**Figure 3:**
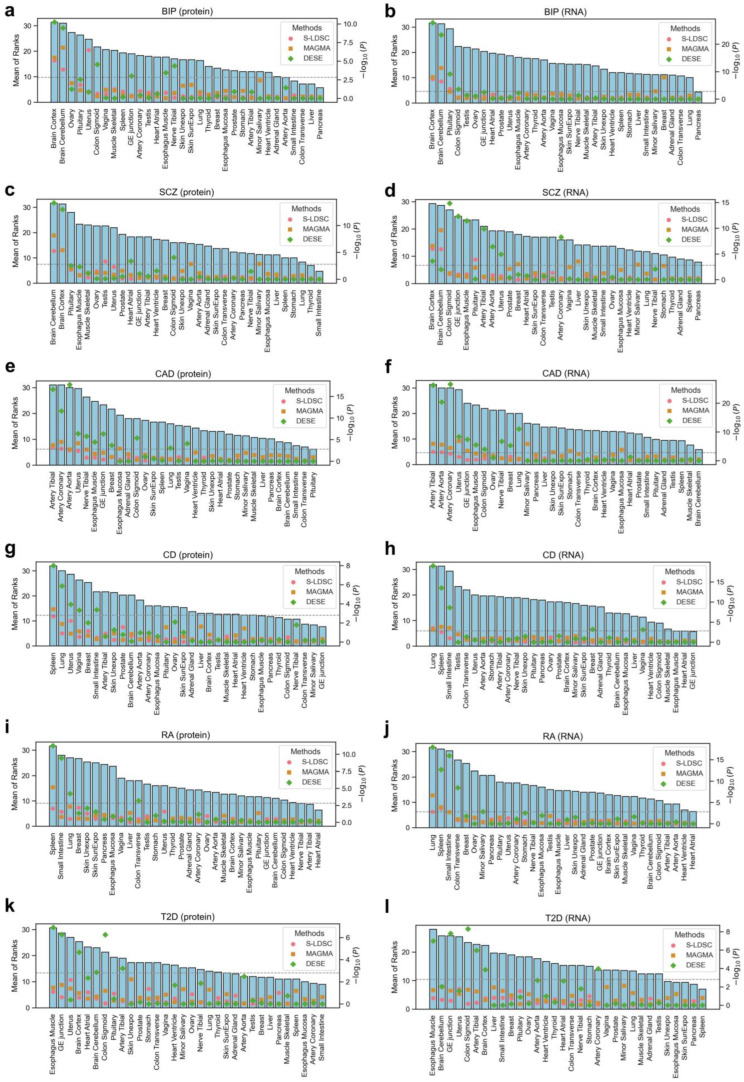
Disease-associated tissues for six complex diseases. Each row of paired subplots represents a different disease: (**a**,**b**) Bipolar disorder; (**c**,**d**) Schizophrenia; (**e**,**f**) Coronary artery disease; (**g**,**h**) Crohn’s disease; (**i**,**j**) Rheumatoid arthritis; (**k**,**l**) Type 2 diabetes. The first column of subplots (**a**,**c**,**e**,**g**,**i**,**k**) shows the disease-tissue associations estimated using protein abundance data, while the second column (**b**,**d**,**f**,**h**,**j**,**l**) shows those estimated using RNA expression data. In each subplot, scatter points with three different shapes represent the −log10 transformed *p*-values obtained from three different methods, with their magnitudes indicated on the right y-axis. The gray horizontal dashed line represents the Bonferroni-corrected significance threshold (0.05/32). The bar plots display the average ranks across the three methods, with their magnitudes indicated on the left y-axis.

We identified disease-associated tissues for six representative complex diseases using three different methods, separately incorporating protein abundance and RNA expression data (Figure 3). The association strength was determined by averaging the rankings of the three methods.

Both protein-based and RNA-based analyses identified the cerebral cortex and cerebellum as the top two associated tissues for bipolar disorder (Figure 3a,b) and schizophrenia (Figure 3c,d). Interestingly, protein-based analysis ranked the cerebellum as the top associated tissue for schizophrenia, highlighting its role in schizophrenia at the protein level [21,22].

For coronary artery disease, both protein-based and RNA-based analyses consistently identified three arterial tissues as the top-ranked associated tissues (Figure 3e,f). Notably, protein-based analysis precisely ranked the coronary artery as the most associated tissue, whereas RNA-based analysis ranked the tibial artery as the top associated tissue.

For the immune diseases Crohn’s disease (Figure 3g,h) and rheumatoid arthritis (Figure 3i,j), both analyses identified immune-related organs such as the spleen and organs with high immune cell distribution (e.g., lung [23] and small intestine [24]) as the most associated tissues. Interestingly, increasing evidence highlights a strong link between rheumatoid arthritis (RA) and lung involvement, particularly interstitial lung disease (ILD), which affects up to 60% of patients [25,26,27]. Notably, pulmonary abnormalities and autoimmune activity may precede joint symptoms, suggesting the lung as a potential site of disease initiation [28,29]. This supports our observed lung signal and implies a direct role of pulmonary immune responses in RA pathogenesis.

In the analysis of type 2 diabetes (T2D), both RNA- and protein-based methods consistently identified esophagus muscle as the most significantly associated tissue (Figure 3k,l). While this finding may initially seem unexpected, the esophagus muscle is a smooth muscle-rich tissue, and muscle-related tissues are known to play important roles in glucose metabolism and insulin resistance [30,31]. Notably, the expression datasets used in our study do not include adipose tissues, which are key metabolic organs and have been frequently implicated in T2D etiology [31]. The absence of adipose tissue data likely limited our ability to detect adipose-related signals. Supporting this interpretation, previous studies have reported that adipose tissue shows the strongest enrichment for T2D heritability, followed by skeletal and smooth muscle tissues [32,33]. Therefore, our results are consistent with known biology to the extent permitted by tissue availability, and the observed esophageal signal may reflect the contribution of muscle-related gene expression to T2D pathogenesis.

Overall, both protein- and RNA-based analyses effectively identified disease-associated tissues, though in certain cases, the emphasis on specific associated tissues differed between the two approaches. Notably, in a certain case (i.e., coronary artery disease), the protein-based approach demonstrated greater sensitivity in capturing disease-relevant tissues, highlighting its distinct advantage over RNA-based analysis.

### 3.3. Evaluation of Disease-Associated Genes

**Figure 4:**
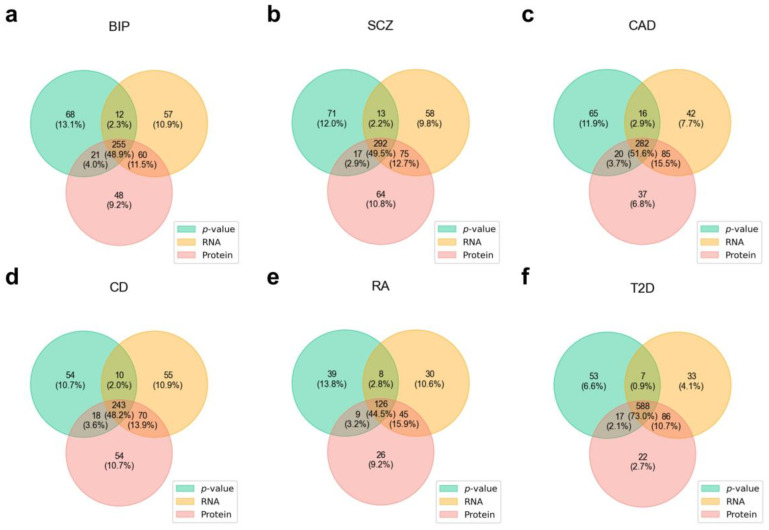
Comparison of disease-associated genes identified by different methods. Venn diagram comparing the disease-associated genes identified by three different fine-mapping methods for six complex diseases: (**a**) Bipolar disorder; (**b**) Schizophrenia; (**c**) Coronary artery disease; (**d**) Crohn’s disease; (**e**) Rheumatoid arthritis; (**f**) Type 2 diabetes. *p*-value refers to the conditional association analysis guided by association *p*-values, RNA represents the DESE method analysis based on RNA-level expression data, and Protein refers to the DESE method analysis based on protein abundance data (see details in Section 2.5: Materials and Method).

The DESE method performs gene fine-mapping based on conditional ECS, where the original conditional ECS prioritizes genes for conditional analysis using gene-based association *p*-values, while DESE prioritizes genes using their specific expression in disease-associated tissues, thereby improving accuracy [2]. Here, we applied DESE using both protein abundance and RNA expression data, along with the original conditional ECS analysis, and then compared the results (Figure 4). The genes identified through protein-based fine-mapping showed more overlap with those identified using RNA-based fine-mapping than with those identified using the *p*-value-based method. For instance, in schizophrenia, the protein-based method shared 367 genes with the RNA-based method, whereas it shared 307 genes with the *p*-value-based method. However, notable differences were also observed between the protein- and RNA-based methods. For example, in schizophrenia, the protein-based method identified 81 unique genes that were not detected using RNA-based fine-mapping. The complete results of gene fine-mapping based on protein and RNA expression can be found in Supplementary Table S1.

**Figure 5:**
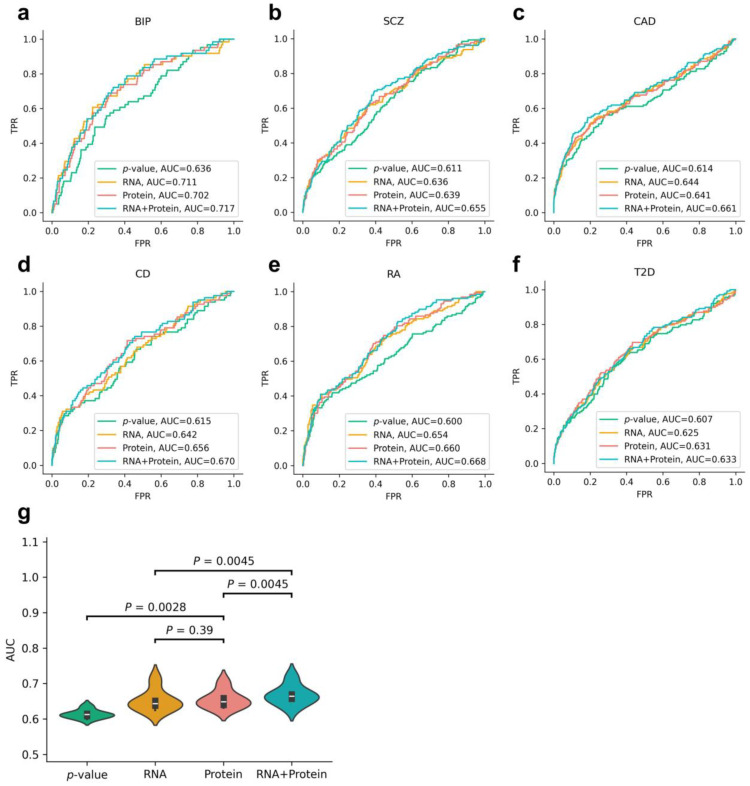
Evaluation of disease-associated genes obtained using different methods. Panels (**a**–**f**) show the ROC curves of associated genes obtained from the four fine-mapping gene analysis methods. Panel (**g**) displays a violin plot of AUC values for six diseases, with *p*-values derived from paired two-tailed *t*-tests.

To assess which approach provides more accurate disease-associated genes, we evaluated those approaches based on PubMed literature validation (Figure 5). Across six diseases, both the protein-based and RNA-based methods outperform the *p*-value-based method in terms of AUC values (Figure 5a–f). For instance, in schizophrenia, the AUC value for the protein-based method was 0.639; for the RNA-based method, it was 0.636; and for the *p*-value-based method, it was 0.611 (Figure 5b). In four out of six diseases (i.e., SCZ, CD, RA, and T2D), the AUC values of the protein-based method were higher than those of the RNA-based method, whereas the opposite was observed for the remaining two diseases (i.e., BIP and CAD). We used paired two-tailed *t*-tests to evaluate whether there were differences in the AUC values of those methods across the six diseases (Figure 5g). The protein-based method was significantly higher than the *p*-value-based method (*p* = 0.0028), but there was no significant difference compared to the RNA-based method (*p* = 0.39).

To further investigate whether integrating the RNA- and protein-based results could improve prediction accuracy, we calculated the geometric mean of the *p*-values from the two approaches as an integrated *p*-value (labeled as the “RNA + Protein” method; see Section 2.5: Materials and Methods for details). We found that, across all six diseases, the integrated method consistently achieved higher AUC values than using either omics layer alone. Moreover, the integrated method showed significantly better performance than the RNA-based (*p* = 0.0045) and protein-based (*p* = 0.0045) methods individually (Figure 5g). These results indicate that incorporating proteomic information significantly improves the estimation of disease-associated genes compared to using RNA alone, highlighting the importance of integrating proteomics in elucidating the pathogenic mechanisms of complex diseases.

### 3.4. Functional Enrichment Analysis of Disease-Associated Genes

**Figure 6:**
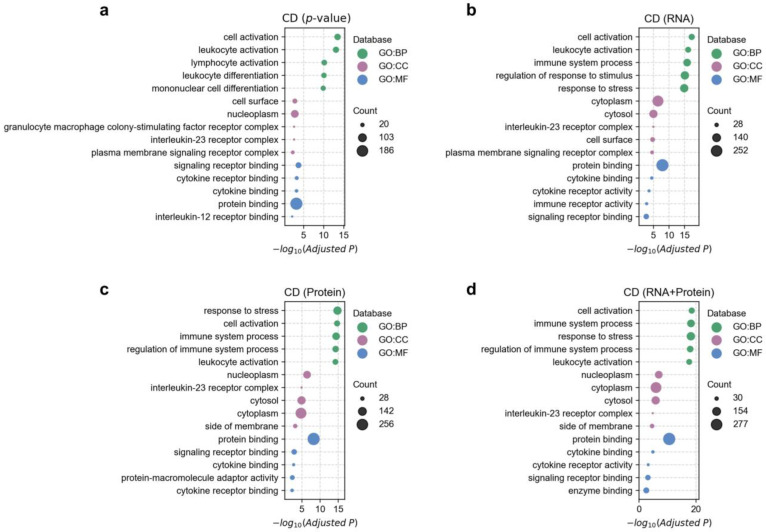
Gene ontology (GO) enrichment analysis of fine-mapped genes implicated in Crohn’s disease (CD). Panels (**a**–**d**) show the GO enrichment results of significantly associated genes (FDR < 0.05) identified by four different fine-mapping strategies (see Materials and Methods Section 2.5 for details). For visualization simplicity, only the top five most significantly associated terms from each database are shown. The bubble color represents different databases, the bubble size indicates the number of overlapping genes between the term and disease-associated genes, and the x-axis represents the negative logarithm (base 10) of the adjusted *p*-value.

We further performed Gene Ontology (GO) functional enrichment analysis on the disease-associated genes (Figure 6 and Supplementary Figures S1–S5). Overall, disease-associated genes identified through protein-based fine-mapping were enriched in biologically relevant terms. For example, in Crohn’s disease, the enriched terms were predominantly immune-related (Figure 6c), such as cell activation (adjusted *p* = 2.1 × 10^−15^) and leukocyte activation (adjusted *p* = 6.1 × 10^−15^). In bipolar disorder, the enriched terms included synapse and ion channel-related terms (Supplementary Figure S1c), such as chemical synaptic transmission (adjusted *p* = 2.1 × 10^−6^), synapse (adjusted *p* = 4.5 × 10^−12^), and calcium ion transmembrane transporter activity (adjusted *p* = 0.001). For coronary artery disease, the enriched terms (Supplementary Figure S3c) included circulatory system development (adjusted *p* = 1.1 × 10^−13^), lipoprotein particle [34] (adjusted *p* = 1.2 × 10^−7^), and cholesterol transfer activity [35] (adjusted *p* = 0.001), which are consistent with previous studies [34,35].

Next, we compared the GO enrichment differences of associated genes identified by different fine-mapping strategies. Overall, the enriched GO terms identified by all methods were largely similar and consistent with known biological knowledge (Figure 6 and Supplementary Figures S1–S5). However, several notable differences emerged. First, in five out of the six diseases (except RA), the most significantly enriched GO terms identified by either the RNA-based or protein-based methods had smaller *p*-values than those identified by the *p*-value-based method. This indicates the added value of incorporating additional omics layers in estimating associated genes, consistent with the earlier gene-level evaluation. Second, the protein-based method generally yielded slightly higher *p*-values for the top-enriched terms compared to the RNA-based method. Third, the *p*-values of the top enriched GO terms identified by the integrated method were very similar to those obtained using the RNA-based method across most diseases. However, in Crohn’s disease (CD), the top enriched term from the integrated method had a markedly smaller *p*-value than that from the RNA-based method (Figure 6b,d); specifically, for the term cell activation, the adjusted *p*-value was 3.2 × 10^−19^ for the integrated method and 4.7 × 10^−18^ for the RNA-based method.

**Figure 7:**
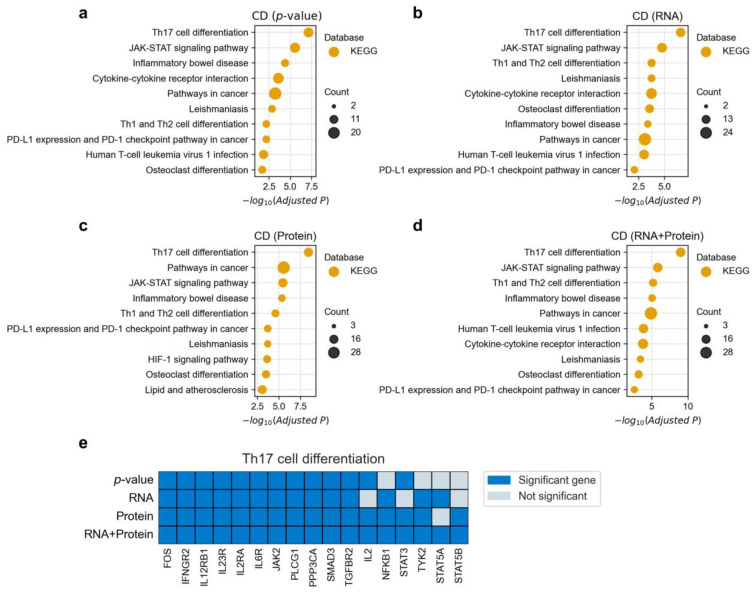
KEGG pathway enrichment analysis of fine-mapped genes implicated in Crohn’s disease (CD). Panels (**a**–**d**) show the KEGG enrichment results of significantly associated genes (FDR < 0.05) identified by four different fine-mapping strategies (see Materials and Methods Section 2.5 for details). For visualization simplicity, only the top ten most significantly associated terms (adjusted *p* < 0.1) from each database are shown. The bubble size indicates the number of overlapping genes between the term and disease-associated genes, and the x-axis represents the negative logarithm (base 10) of the adjusted *p*-value. Panel (**e**) shows CD-associated genes identified within the Th17 cell differentiation pathway using four different strategies. The y-axis represents the four fine-mapping strategies, and the x-axis represents genes in the pathway. Dark blue indicates significant association with CD, while light blue indicates no significant association.

Given the broad functional scope of GO terms, we further examined the KEGG pathway enrichment results for the associated genes (Figure 7 and Supplementary Figures S6–S10). Here, we use Crohn’s disease (CD) as an example. All four methods identified the Th17 cell differentiation pathway as the most significantly enriched pathway, with the integrated method yielding the smallest *p*-value (adjusted *p* = 7.0 × 10^−8^). Th17 cells are a distinct subset of pro-inflammatory T helper cells that play a critical role in mucosal immunity and inflammation [36]. Substantial evidence has linked dysregulated Th17 responses to the pathogenesis of CD [37]. Previous studies have demonstrated elevated levels of Th17 cells and their cytokines (e.g., IL-17A and IL-22) in the intestinal mucosa of CD patients [38,39], implicating this pathway as a strong and well-established contributor to disease pathology. We further examined the CD-associated genes identified within the Th17 pathway by each of the four methods (Figure 7e). The integrated method identified the largest number of genes (17), followed by the protein-based (16), RNA-based (14), and *p*-value-based (13) methods. Notably, *IL2* and *STAT3* were identified by the protein-based method but missed by the RNA-based method. These two genes play pivotal roles in Th17 cell differentiation as follows: *IL2* regulates the balance between Treg and Th17 cells, and its dysregulation can shift immune responses toward a pro-inflammatory state [40]. *STAT3* is a central transcription factor essential for Th17 differentiation, mediating the signaling of cytokines such as IL-6 and IL-23 (Supplementary Figure S11). Moreover, the integrated method uniquely identified both *STAT5A* and *STAT5B* as significantly associated genes. These genes are known to negatively regulate Th17 differentiation and contribute to T cell homeostasis, highlighting their key role within this pathway [41]. Together, these findings underscore the importance of protein-level information and demonstrate that integrating multi-omics evidence can enhance the detection of functionally relevant disease-associated genes.

### 3.5. Unique Disease-Gene Associations Identified by Protein-Based Fine-Mapping

We next focused on disease-gene associations that were identified by protein-based analyses but not captured by RNA-based analyses. Specifically, we examined genes that exhibited high tissue-specific protein expression in disease-relevant tissues but low tissue-specific RNA expression, which would likely be overlooked in RNA-based fine-mapping (Table 2; full results are provided in Supplementary Table S2).

For bipolar disorder, *CREB1* was exclusively identified by the protein-based fine-mapping method (*p* = 7.9 × 10^−5^). Its tissue-specific expression percentile in the cerebellum was 86% at the protein level but only 42% at the RNA level. *CREB1* encodes a transcription factor involved in calmodulin-induced pathways [42] and has been linked to bipolar disorder in 15 PubMed articles. Another gene, *NME2*, was found to be significantly associated with bipolar disorder based on protein-based fine-mapping (*p* = 8.6 × 10^−6^) but not by RNA-based fine-mapping (*p* = 1). In the cerebellum, tissue-specific expression of *NME2* ranked in the 78th percentile at the protein level, compared to only the 2nd percentile at the RNA level. *NME2* encodes a protein that plays a key role in synthesizing nucleoside triphosphates other than ATP [42]. Although no previous literature has directly linked *NME2* to bipolar disorder, our findings suggest its potential role at the protein level in the disease pathogenesis.

For coronary artery disease (CAD), *SMARCA4* was identified as a significantly associated gene only through the protein-based approach (*p* = 3.3 × 10^−23^). This may be due to its high tissue-specific expression at the protein level in coronary arteries (81st percentile) while having minimal tissue-specific expression at the RNA level (5th percentile). *SMARCA4* has been linked to CAD in 11 PubMed articles. Similarly, for Crohn’s disease, *STAT3* was identified as a significant associated gene using the protein-based approach (*p* = 9.1 × 10^−5^), whereas the RNA-based approach did not yield statistical significance (*p* = 0.012). *STAT3* has been reported in over 151 PubMed articles as being associated with Crohn’s disease.

In summary, discrepancies between protein and RNA expression levels lead to differences in disease-associated gene identification. Unique disease-associated genes identified through protein-based fine-mapping highlight the necessity of exploring disease mechanisms from a protein-level perspective.

## 4. Discussion

Unlike previous studies that focused on RNA expression, our study integrates protein expression profiles with GWAS data to investigate disease-associated tissues and genes in complex diseases, highlighting the potential role of proteins in disease pathogenesis. Both prior findings [7,8,9] and our results indicate a substantial discrepancy between protein and RNA expression levels, which underscores the necessity of studying proteins in disease mechanisms. To this end, we systematically compared the effectiveness of protein- and RNA-based approaches in identifying disease-associated tissues and fine-mapped genes using paired expression profile data.

At the disease-associated tissue level, both protein- and RNA-based expression analyses were generally effective in identifying disease-associated tissues. However, in certain cases, protein-based analysis appeared more reasonable. For example, the protein-based approach identified the coronary artery as the top-ranked tissue for coronary artery disease (CAD), whereas the RNA-based approach ranked the tibial artery first and the coronary artery second.

At the fine-mapped gene level, there was substantial overlap between genes identified by protein- and RNA-based analyses, but some disease-associated genes were uniquely captured by protein-based analysis. Our computational validation indicated no significant difference in accuracy between the genes identified by the two approaches. However, integrating the RNA- and protein-based results significantly improved the estimation of disease-associated genes, underscoring the importance of incorporating protein-level evidence in the interpretation of complex disease mechanisms. Functional enrichment analysis of the fine-mapped genes revealed biologically plausible pathways. Interestingly, in certain diseases such as Crohn’s disease (CD), integrating RNA- and protein-based analysis led to more significant enrichment of disease-relevant pathways, thereby facilitating a deeper understanding of the underlying pathogenic mechanisms. Notably, we identified unique disease-gene associations based on protein expression, where parts of these genes exhibited low RNA-specific expression but high protein-specific expression in disease-associated tissues. Since previous studies have primarily focused on RNA expression, it is crucial to pay closer attention to these protein-identified disease-associated genes that were overlooked at the RNA level.

Nevertheless, comprehensive and accurate quantification of proteins remains a major technical challenge. Compared to RNA expression data, publicly available protein expression data are still relatively scarce. Future advancements in protein quantification depth and precision will be essential for gaining deeper insights into the pathogenesis of complex diseases.

We employed PubMed literature evidence as a proxy for gene-level validation, a common strategy in gene prioritization studies [2,43]. While this method offers a standardized and interpretable benchmark, it has limitations, including a bias toward well-studied genes and the possibility that the literature mentioned may not reflect causal relevance. As such, the literature-based validation should be interpreted with caution and viewed as supportive rather than definitive evidence of biological importance.

Although our analysis utilizes cross-sectional transcriptomic and proteomic data to explore disease-associated gene expression patterns across tissues, it does not account for temporal dynamics. This limitation is particularly relevant for psychiatric disorders, which often exhibit distinct age-of-onset patterns and progression trajectories [44]. Capturing such temporal variation would require access to time-resolved proteomic data. Incorporating longitudinal proteomics in future studies may provide deeper insights into dynamic regulatory mechanisms and the temporal evolution of disease. Our study is based on bulk-level RNA and protein expression profiles, which reflect averaged signals across heterogeneous cell populations within each tissue. This limitation may obscure cell-type-specific regulatory patterns that are relevant for disease mechanisms. Future integration of single-cell or spatially resolved transcriptomic and proteomic data would help deconvolve these signals and enable a more precise understanding of the cellular context underlying tissue-level associations.

It is important to note that proteomic data are subject to detection bias, favoring proteins with higher abundance. This technical limitation may reduce the coverage of low-abundance but functionally important proteins, such as certain transcription factors or signaling molecules. As a result, some disease-relevant signals captured at the RNA level may not be detectable at the protein level, potentially contributing to the differences observed between RNA- and protein-based mapping results. This inherent bias should be considered when interpreting the relative utility of transcriptomic versus proteomic data in disease gene prioritization.

## 5. Conclusions

In this study, we systematically integrated proteomic data with genome-wide association studies (GWASs) to identify disease-associated tissues and fine-map susceptibility genes across six major complex diseases. We demonstrated that protein abundance, while moderately correlated with RNA expression, provides distinct and biologically meaningful insights. Proteomic data not only improved the accuracy of tissue prioritization in the specific case—for example, correctly identifying the coronary artery as the most relevant tissue in coronary artery disease—but also revealed unique disease-gene associations that RNA-based analyses overlooked.

By comparing RNA-based, protein-based, and integrated gene fine-mapping strategies, we found that integrating proteomic data with RNA data significantly enhances the identification of disease-associated genes and pathways compared to using either alone, as validated by functional enrichment analyses and literature evidence. Our results highlight the indispensable role of proteomics in advancing our understanding of complex disease biology and suggest that future disease-mapping efforts should incorporate proteomic information to uncover mechanisms that may be invisible through transcriptomic analysis alone.

## Figures and Tables

**Table 1:** Summary of GWAS datasets for six representative complex diseases.

Abbreviation	Disease Name	PMID	Source	Sample Size
BIP	Bipolar disorder	34002096	PGC	413,466
SCZ	Schizophrenia	35396580	PGC	320,404
CAD	Coronary artery disease	29212778	GWAS Catalog	296,525
CD	Crohn’s disease	28067908	GWAS Catalog	40,266
RA	Rheumatoid arthritis	24390342	GWAS Catalog	57,284
T2D	Type 2 diabetes	39024449	GWAS Catalog	432,648

PMID refers to the PubMed ID of the source publication for each GWAS dataset. The source indicates where the GWAS summary statistics were obtained. Data from the Psychiatric Genomics Consortium (PGC) can be accessed at https://pgc.unc.edu/for-researchers/download-results/ (accessed on 14 March 2024), and data from the GWAS Catalog are available at https://www.ebi.ac.uk/gwas/ (accessed on 19 February 2025).

**Table 2:** Examples of unique disease-gene associations identified based on protein abundance profiles.

Disease	Gene	P (Protein)	P (RNA)	PubMed Count	Associated Tissue	Rank (Protein)	Rank (RNA)
BIP	*CREB1*	7.93 × 10^−5^	0.054	15	BrainCerebellum	0.864	0.424
BIP	*NME2*	8.56 × 10^−6^	1.000	0	BrainCerebellum	0.780	0.019
SCZ	*HSPD1*	1.05 × 10^−14^	0.115	11	BrainCerebellum	0.797	0.354
SCZ	*CENPA*	1.20 × 10^−6^	0.268	1	BrainCortex	0.876	0.185
CAD	*SMARCA4*	3.29 × 10^−23^	0.021	11	ArteryCoronary	0.812	0.052
CAD	*TNRC6B*	4.84 × 10^−5^	0.073	0	ArteryCoronary	0.943	0.013
CD	*STAT3*	9.13 × 10^−5^	0.012	151	Spleen	0.799	0.593
CD	*RAD50*	4.95 × 10^−13^	0.011	1	Spleen	0.809	0.010
RA	*ARCN1*	4.14 × 10^−5^	1.000	53	Spleen	0.720	0.161
RA	*SMARCC2*	4.71 × 10^−5^	1.000	0	Spleen	0.867	0.215
T2D	*CYP17A1*	4.08 × 10^−9^	1.000	7	EsophagusMuscle	0.818	0.296
T2D	*GPN1*	8.61 × 10^−6^	1.000	0	EsophagusMuscle	0.818	0.194

P (Protein) and P (RNA) represent the conditional gene-based association *p*-values obtained from protein- and RNA-based analyses, respectively. PubMed count indicates the number of publications in which the disease and gene co-appear. Rank (Protein) and Rank (RNA) denote the normalized tissue-specific expression ranks for protein and RNA in disease-associated tissues, with higher values indicating stronger tissue-specific expression.

## Data Availability

All data are available on request.

## Supplementary Materials

The following supporting information can be downloaded at: https://www.mdpi.com/article/10.3390/biology14050554/s1, Figures S1–S5: GO enrichment analysis of fine-mapped genes in BIP, SCZ, CAD, RA, and T2D; Figures S6–S10: KEGG enrichment analysis of fine-mapped genes in BIP, SCZ, CAD, RA, and T2D; Figure S11: KEGG pathway map of Th17 cell differentiation; Table S1: The complete results of gene fine-mapping based on protein and RNA expression; Table S2: The complete results of unique disease-gene associations identified based on protein expression.

## Abbreviations

The following abbreviations are used in this manuscript:

GWAS	genome-wide association study
RNA	ribonucleic acid
GTEx	genotype-tissue expression
CI	confidence interval
ROC	receiver operating characteristic
AUC	area under the curve
HGNC	HUGO Gene Nomenclature Committee
LD	linkage disequilibrium
MHC	major histocompatibility complex
BIP	bipolar disorder
SCZ	schizophrenia
CAD	coronary artery disease
CD	Crohn’s disease
RA	rheumatoid arthritis
T2D	type 2 diabetes
FDR	false discovery rate
API	application programming interface
GO	Gene Ontology
PGC	Psychiatric Genomics Consortium
