# Supplementary material for: Integrating Proteomics and GWAS to Identify Key Tissues and Genes Underlying Human Complex Diseases
Source: Biology (Basel). 2025 May 16;14(5):554. doi: 10.3390/biology14050554 (PMC12109507; doi:10.3390/biology14050554)
Supplement: Supplementary file 1 [file biology-14-00554-s001.zip › Supplementary Figures.pdf]

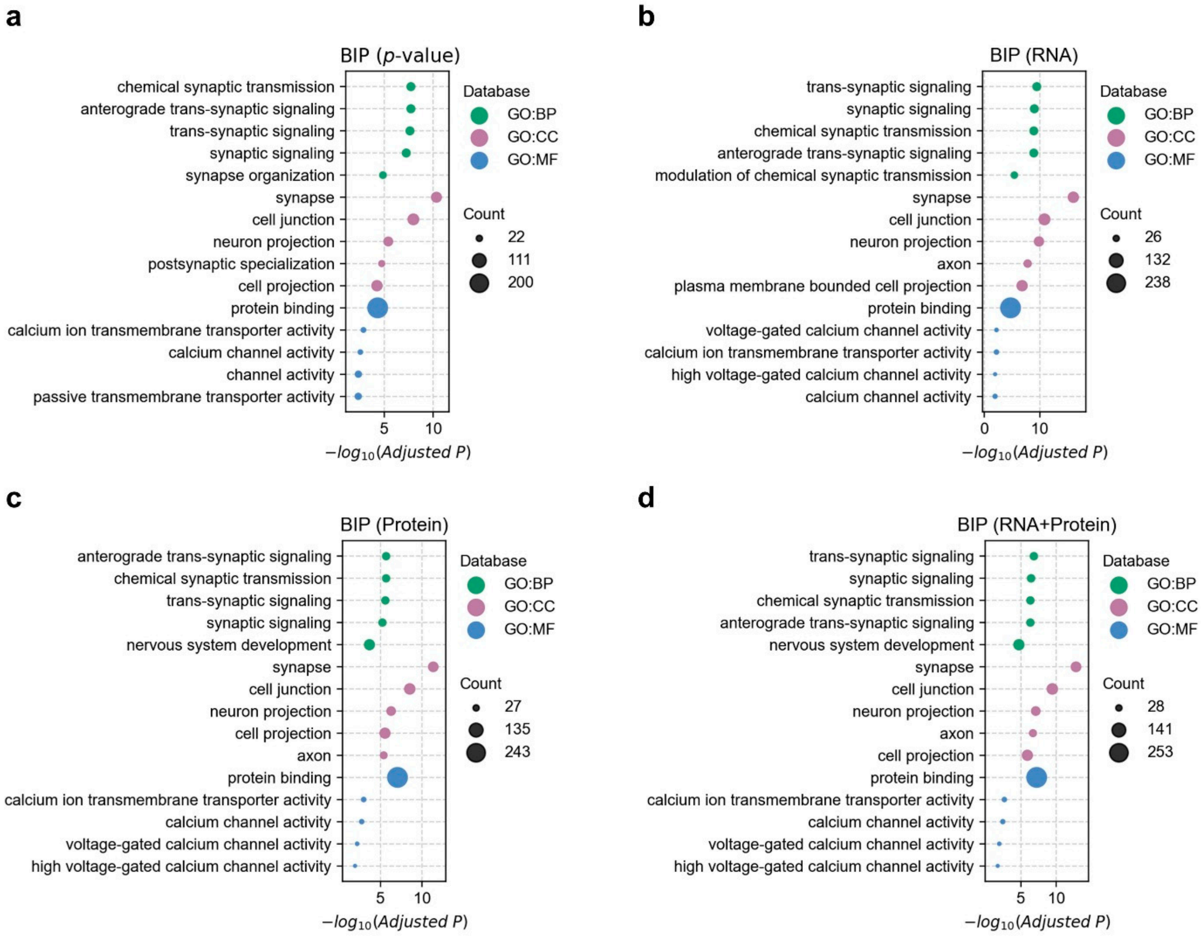

Figure S1. Gene ontology (GO) enrichment analysis of fine-mapped genes implicated in bipolar disorder (BIP). Panels (a–d) show the GO enrichment results of significantly associated genes (FDR<0.05) identified by four different fine-mapping strategies (see Methods section 2.5 for details). For visualization simplicity, only the top five most significantly associated terms from each database are shown. The bubble color represents different databases, the bubble size indicates the number of overlapping genes between the term and disease-associated genes, and the x-axis represents the negative logarithm (base 10) of the adjusted p-value.

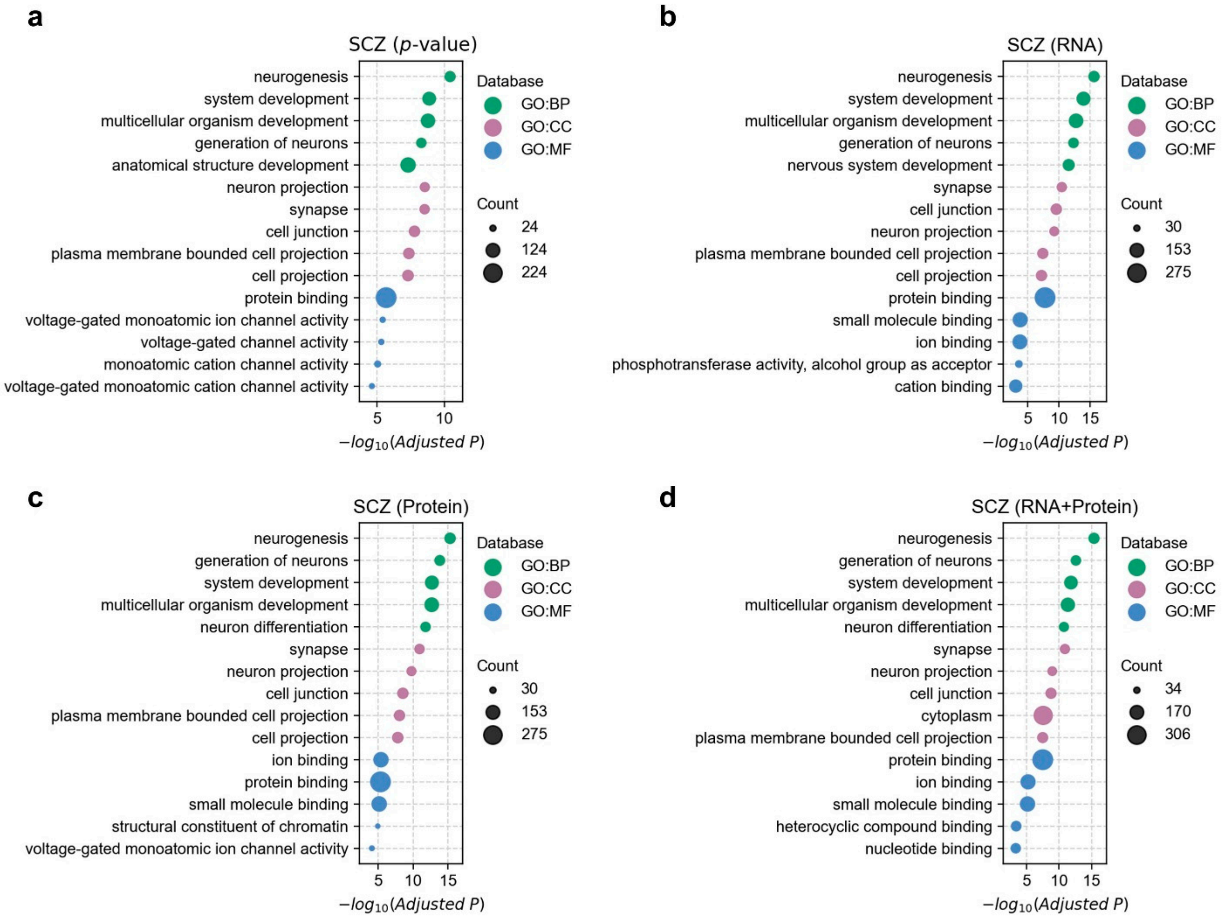

Figure S2. Gene ontology (GO) enrichment analysis of fine-mapped genes implicated in schizophrenia (SCZ). Panels (a–d) show the GO enrichment results of significantly associated genes (FDR<0.05) identified by four different fine-mapping strategies (see Methods section 2.5 for details). For visualization simplicity, only the top five most significantly associated terms from each database are shown. The bubble color represents different databases, the bubble size indicates the number of overlapping genes between the term and disease-associated genes, and the x-axis represents the negative logarithm (base 10) of the adjusted p-value.

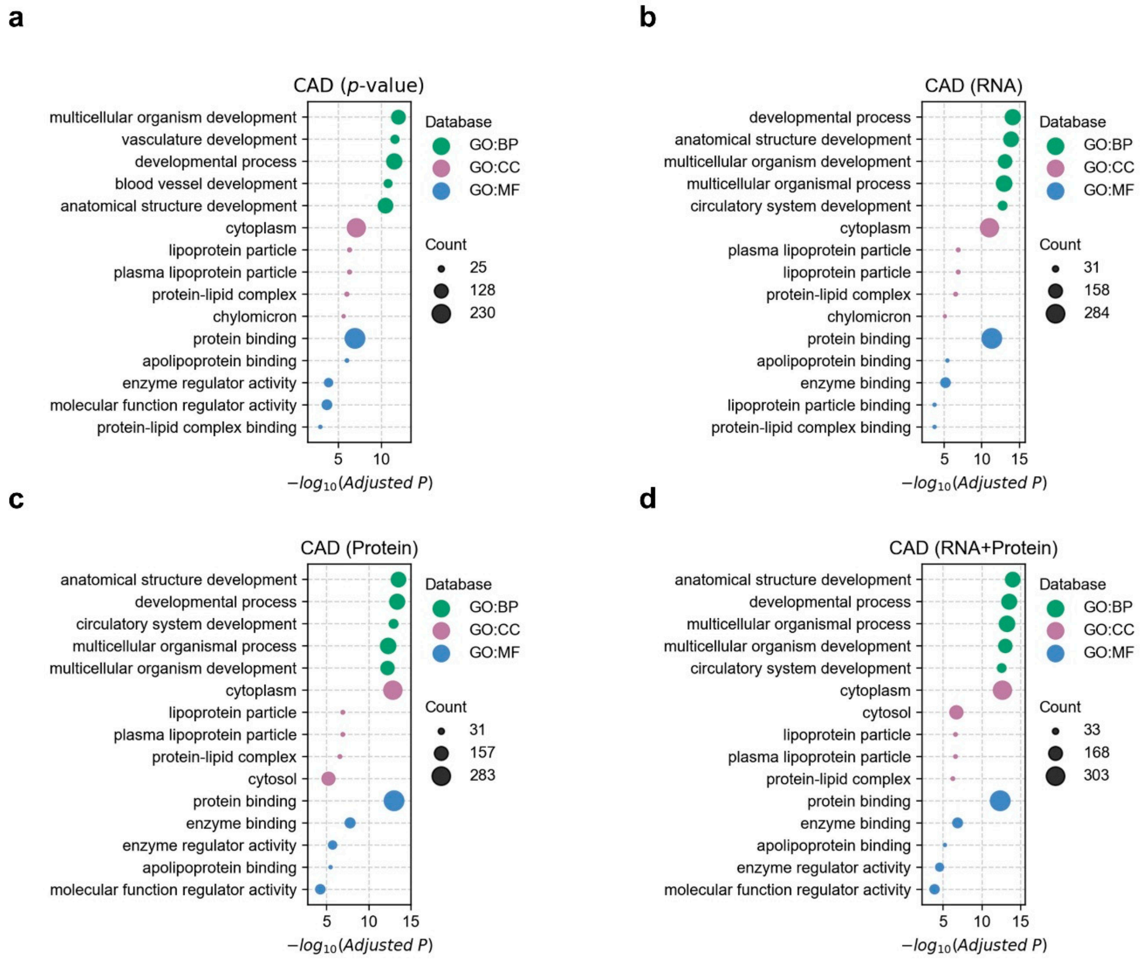

Figure S3. Gene ontology (GO) enrichment analysis of fine-mapped genes implicated in coronary artery disease (CAD). Panels (a–d) show the GO enrichment results of significantly associated genes (FDR<0.05) identified by four different fine-mapping strategies (see Methods section 2.5 for details). For visualization simplicity, only the top five most significantly associated terms from each database are shown. The bubble color represents different databases, the bubble size indicates the number of overlapping genes between the term and disease-associated genes, and the x-axis represents the negative logarithm (base 10) of the adjusted p-value.

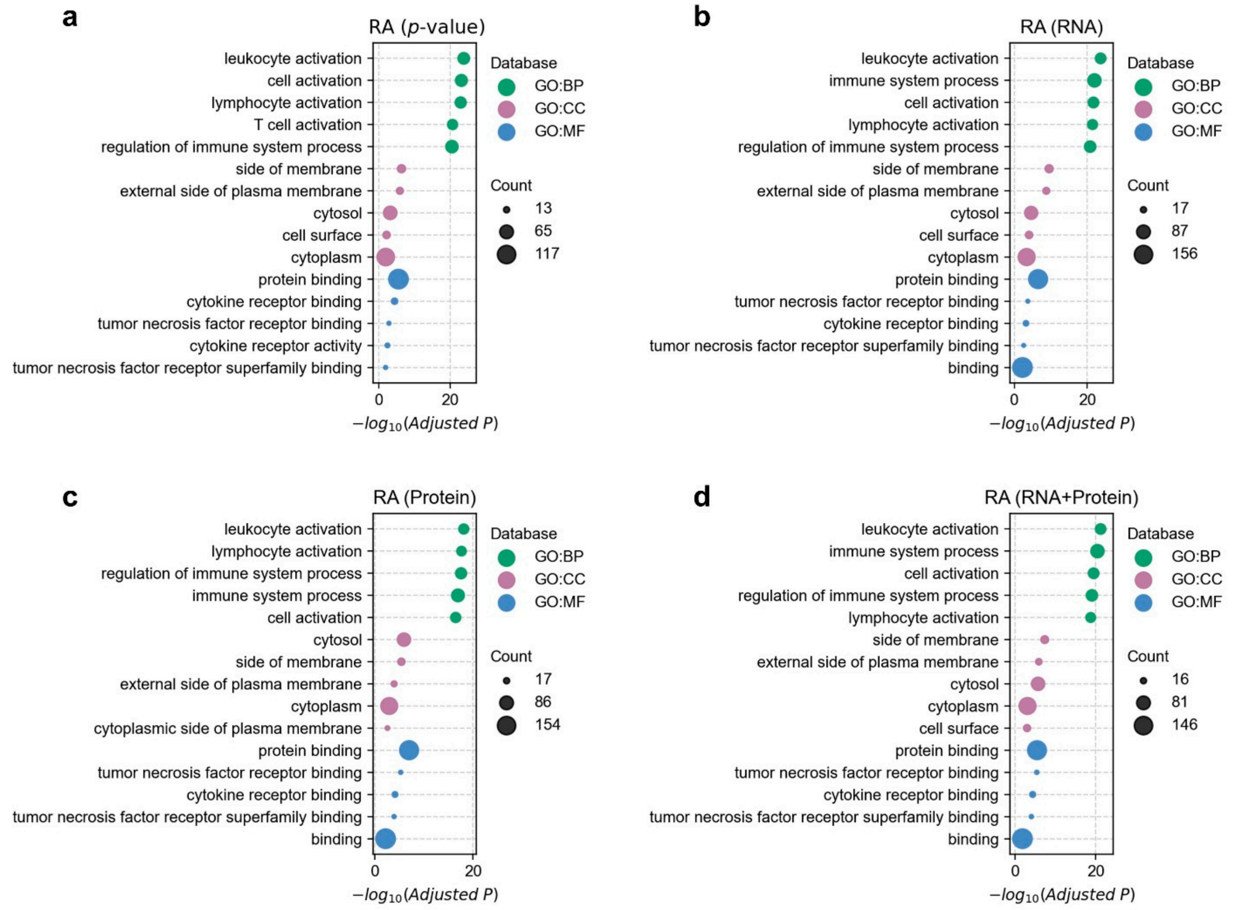

Figure S4. Gene ontology (GO) enrichment analysis of fine-mapped genes implicated in rheumatoid arthritis (RA). Panels (a–d) show the GO enrichment results of significantly associated genes (FDR<0.05) identified by four different fine-mapping strategies (see Methods section 2.5 for details). For visualization simplicity, only the top five most significantly associated terms from each database are shown. The bubble color represents different databases, the bubble size indicates the number of overlapping genes between the term and disease-associated genes, and the x-axis represents the negative logarithm (base 10) of the adjusted p-value.

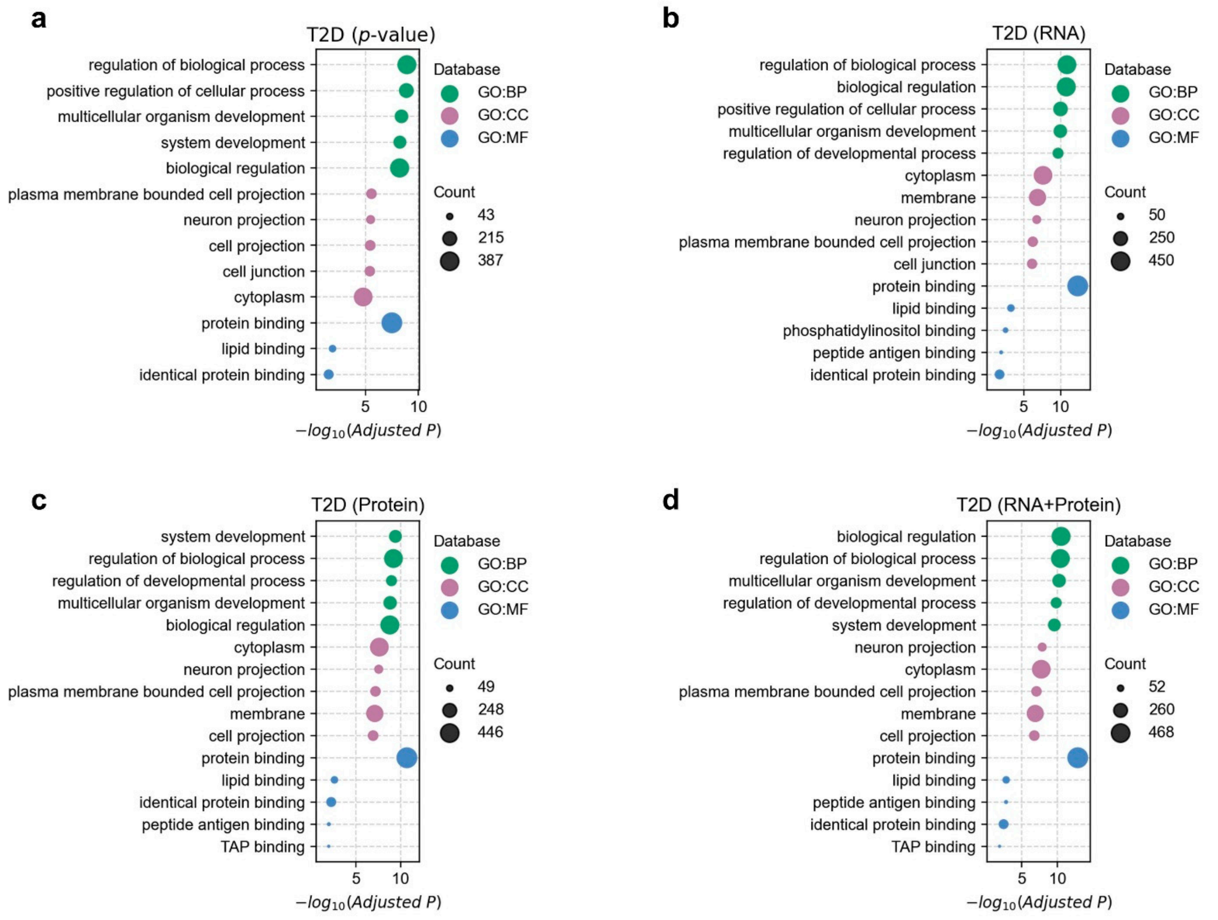

Figure S5. Gene ontology (GO) enrichment analysis of fine-mapped genes implicated in type 2 diabetes (T2D). Panels (a–d) show the GO enrichment results of significantly associated genes (FDR<0.05) identified by four different fine-mapping strategies (see Methods section 2.5 for details). For visualization simplicity, only the top five most significantly associated terms from each database are shown. The bubble color represents different databases, the bubble size indicates the number of overlapping genes between the term and disease-associated genes, and the x-axis represents the negative logarithm (base 10) of the adjusted p-value.

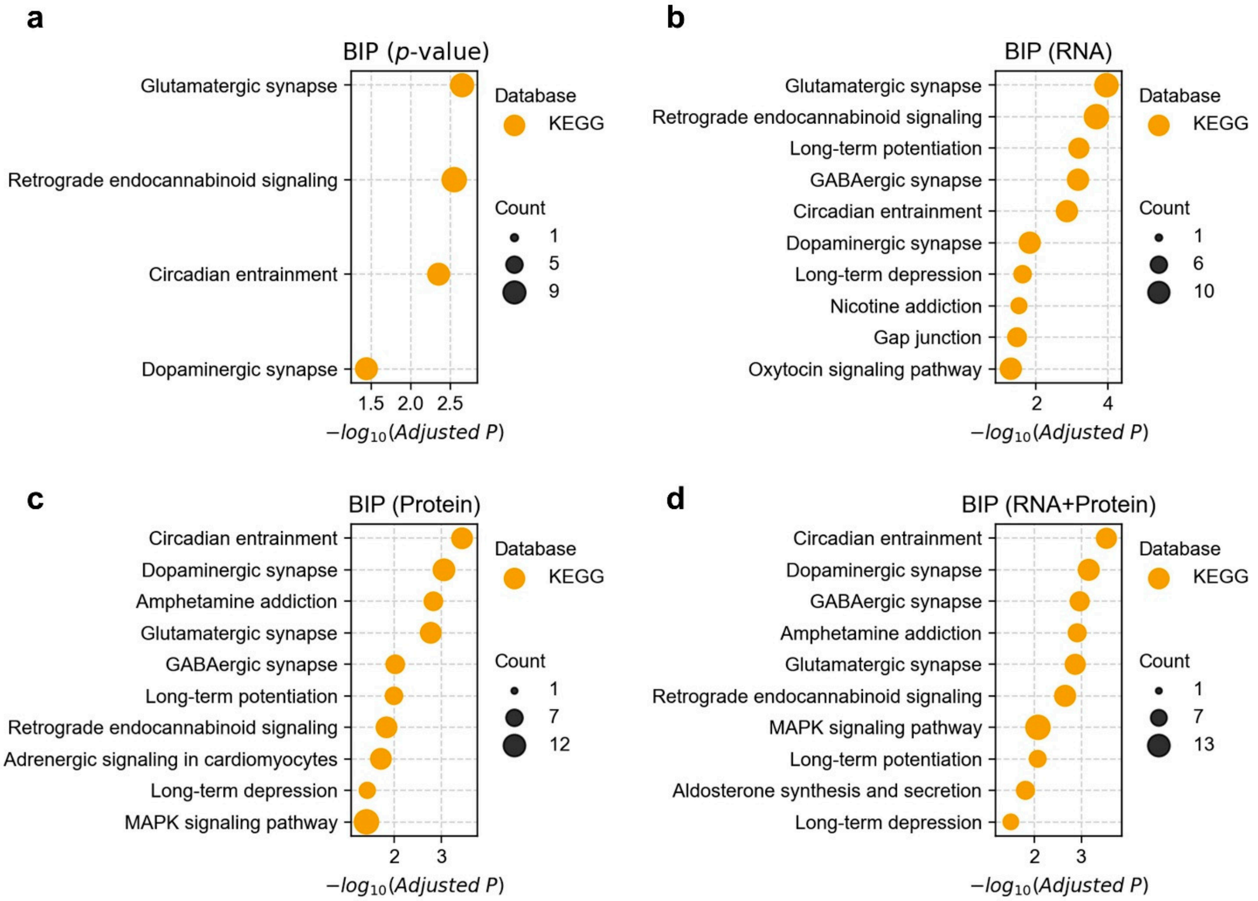

Figure S6. KEGG pathway enrichment analysis of fine-mapped genes implicated in bipolar disorder (BIP). Panels (a–d) show the KEGG enrichment results of significantly associated genes (FDR<0.05) identified by four different fine-mapping strategies (see Methods section 2.5 for details). For visualization simplicity, only the top ten most significantly associated terms (adjusted P<0.1) from each database are shown. The bubble size indicates the number of overlapping genes between the term and disease-associated genes, and the x-axis represents the negative logarithm (base 10) of the adjusted p-value.

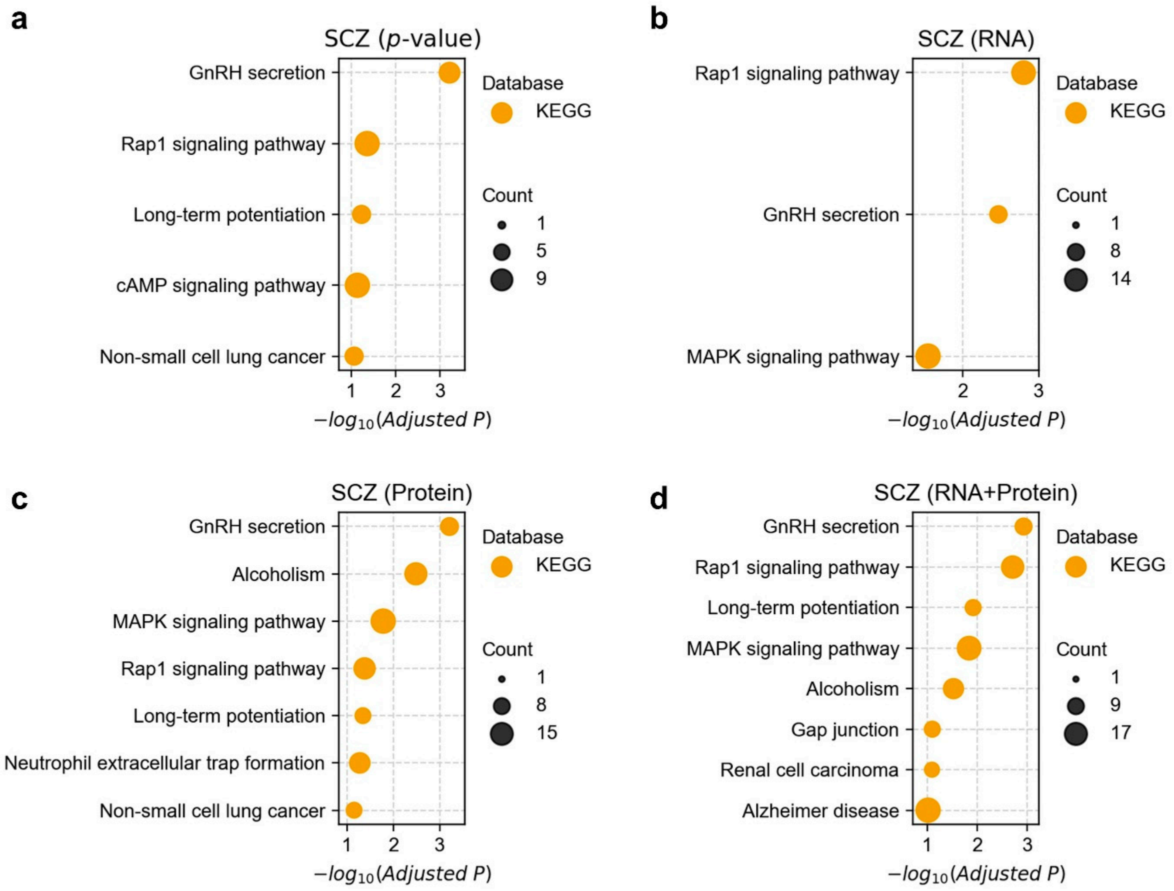

Figure S7. KEGG pathway enrichment analysis of fine-mapped genes implicated in schizophrenia (SCZ). Panels (a–d) show the KEGG enrichment results of significantly associated genes (FDR<0.05) identified by four different fine-mapping strategies (see Methods section 2.5 for details). For visualization simplicity, only the top ten most significantly associated terms (adjusted P<0.1) from each database are shown. The bubble size indicates the number of overlapping genes between the term and disease-associated genes, and the x-axis represents the negative logarithm (base 10) of the adjusted p-value.

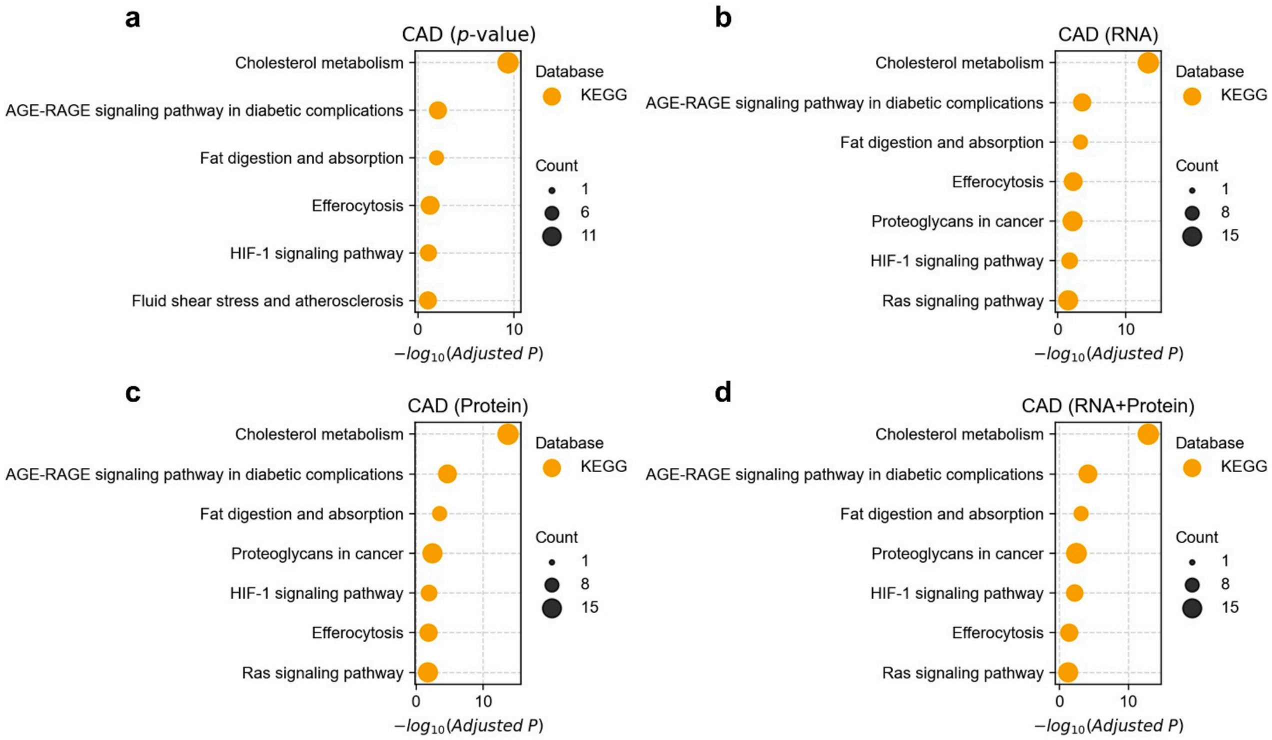

Figure S8. KEGG pathway enrichment analysis of fine-mapped genes implicated in coronary artery disease (CAD). Panels (a–d) show the KEGG enrichment results of significantly associated genes (FDR<0.05) identified by four different fine-mapping strategies (see Methods section 2.5 for details). For visualization simplicity, only the top ten most significantly associated terms (adjusted P<0.1) from each database are shown. The bubble size indicates the number of overlapping genes between the term and disease-associated genes, and the x-axis represents the negative logarithm (base 10) of the adjusted p-value.

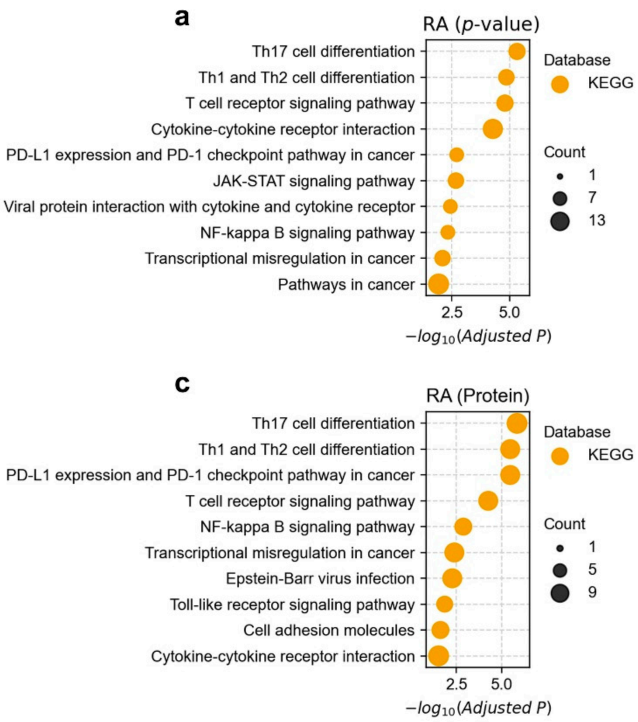

Figure S9. KEGG pathway enrichment analysis of fine-mapped genes implicated in rheumatoid arthritis (RA). Panels (a–d) show the KEGG enrichment results of significantly associated genes (FDR<0.05) identified by four different fine-mapping strategies (see Methods section 2.5 for details). For visualization simplicity, only the top ten most significantly associated terms (adjusted P<0.1) from each database are shown. The bubble size indicates the number of overlapping genes between the term and disease-associated genes, and the x-axis represents the negative logarithm (base 10) of the adjusted p-value.

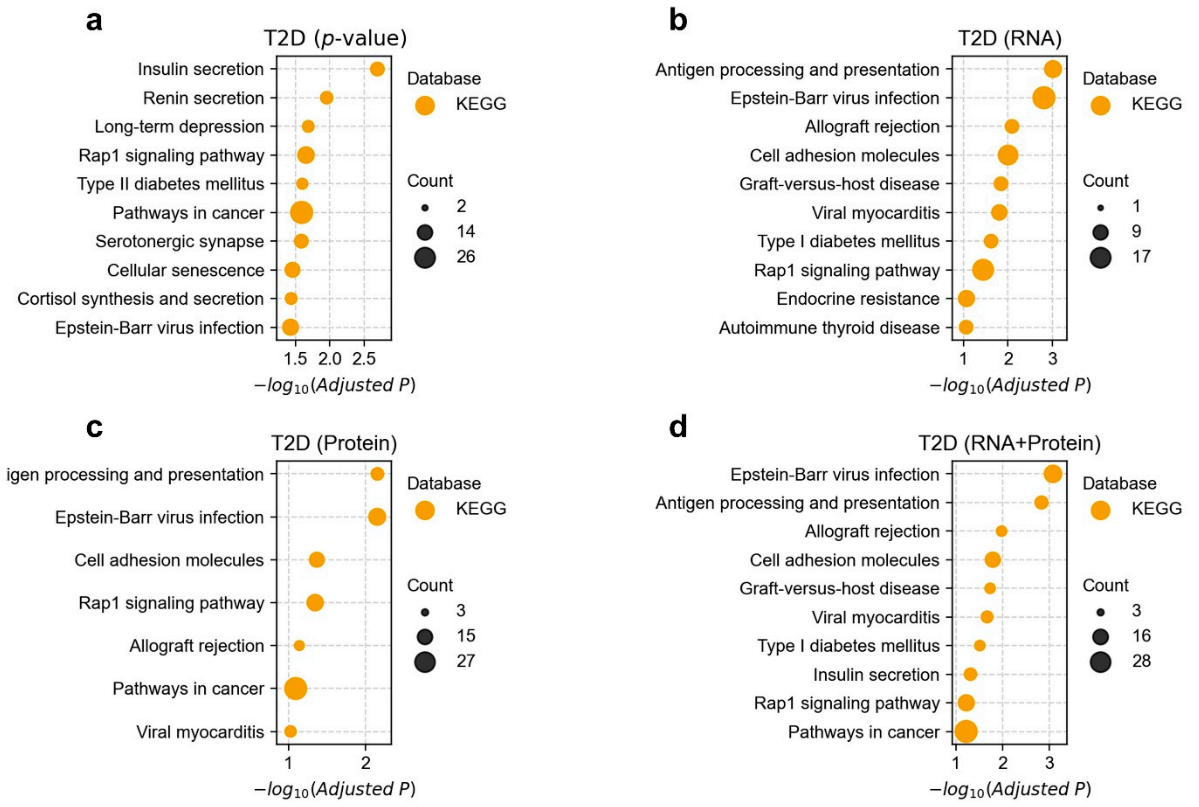

Figure S10. KEGG pathway enrichment analysis of fine-mapped genes implicated in type 2 diabetes (T2D). Panels (a–d) show the KEGG enrichment results of significantly associated genes (FDR<0.05) identified by four different fine-mapping strategies (see Methods section 2.5 for details). For visualization simplicity, only the top ten most significantly associated terms (adjusted P<0.1) from each database are shown. The bubble size indicates the number of overlapping genes between the term and disease-associated genes, and the x-axis represents the negative logarithm (base 10) of the adjusted p-value.

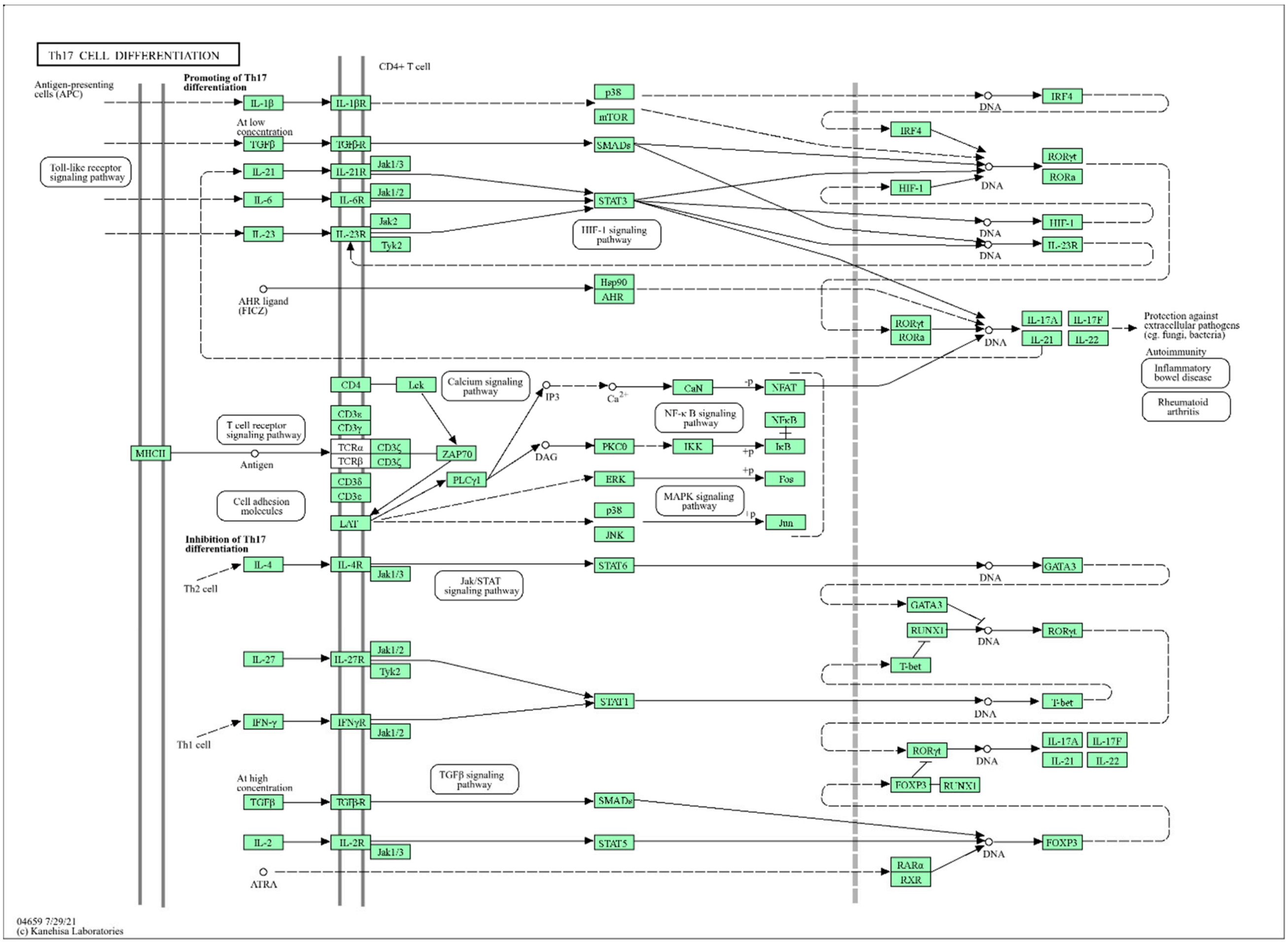

Figure S11. KEGG pathway map of Th17 cell differentiation. The schematic diagram of the Th17 cell differentiation pathway was downloaded from the KEGG database (https://www.kegg.jp/pathway/hsa04659, accessed on 2 May 2025). This pathway has been implicated in the pathogenesis of Crohn's disease and was identified as the top-enriched pathway across multiple fine-mapping strategies in this study.
